# Gender difference in utilization willingness of institutional care among the single seniors: evidence from rural Shandong, China

**DOI:** 10.1186/s12939-017-0577-z

**Published:** 2017-05-12

**Authors:** Yangyang Qian, Jie Chu, Dandan Ge, Li Zhang, Long Sun, Chengchao Zhou

**Affiliations:** 1 School of Public Health, Shandong University, Jinan, 250012 China; 2 Shandong Centre for Disease Control and Prevention, Jinan, 250014 China; 3 School of Public Health, Shandong University; Collaborative Innovation Center of Social Risks Governance in Health, 44 Wen-hua-xi Road, Jinan, Shandong 250012 China

**Keywords:** Institutional care, Willingness, Gender difference, Elderly, Single, Rural, Determinants

## Abstract

**Background:**

Institutional care has become an urgent issue in rural China. Rural single seniors, compared with their counterparts, have lower income and are more vulnerable. Gender is also a significant factor determining long-term institutional care. This study is designed to examine the gender difference towards utilization willingness of institutional care among rural single seniors.

**Methods:**

A total of 505 rural single seniors were included in the analysis. Binary logistic regression model was used to examine the gender difference towards utilization willingness for institutional care, and also to identify the determinants of the utilization willingness for institutional care among rural single male and female seniors.

**Results:**

Our study found that about 5.7% rural single seniors had willingness for institutional care in Shandong, China. Single females were found to be less willing for institutional care than single males in rural areas (OR = 0.19; 95 CI 0.06-0.57). It’s also found that psychological stress was associated with institutionalization willingness in both single males (*P* = 0.045) and single females (*P* = 0.013) in rural China. The rural single seniors who lived alone were found to be more willing for institutional care both in males (*P* = 0.032) and females (*P* = 0.002) compared with those who lived with children or others.

**Conclusions:**

This study found that there was a gender difference towards utilization willingness for institutional care among single seniors in rural China. Factors including psychological stress and living arrangements were determinants of institutionalization willingness both in single males and females. Targeted policies should be made for rural single seniors of different gender.

**Electronic supplementary material:**

The online version of this article (doi:10.1186/s12939-017-0577-z) contains supplementary material, which is available to authorized users.

## Background

For thousands of years, taking care of the elderly by adult children in family was a basic norm in the Confucian doctrine [1]. In recent years, increased geographic mobility and reduced family size due to one-child policy have made more adult children unavailable for elder care [2]. Since China entered the aging society in 1999, the amount of aging population in China has ranked first in the world [3]. The number of Chinese people aged 60 years and above has reached 212.4 million by 2014, which accounted for 15.5% of the general population [4]. It’s estimated that China, with an amount of 98.3 million old people aged 80 or over in 2050, will still be one of those countries which have the greatest numbers of oldest-old [5]. Consequently, institutional care has been strongly promoted to meet older adults’ long-term care needs [6]. According to the Fourth National Health Survey, the percentages of the elderly who were unable to walk (14.9%), who had long-term functional impairment (31.6%), who had difficulty speaking (15.0%), who had moderate vision disability (27.8%) were higher than those in urban areas [7]. Developing long-term care systems for the elderly has become an increasing urgent policy issue in China, especially in the rural areas [8].

According to the population census 2010 in China, single seniors accounted for 29.45% of the general old population [9]. Single seniors refer to those widowed, divorced or unmarried elderly who were unlikely to rely on spouses for support in their daily life activities [10]. Single seniors, compared with those seniors with a spouse, were more vulnerable. Some studies found that those seniors living without a spouse preferred institutional care setting [6]. It is also found that single people were five and a half times as likely, and widowed or divorced people three and a half times as likely as the married to be in an institution [11]. Married older people have about half the probability of nursing home admission of unmarried people, even after controlling for health, demographic, and economic differences [12].

Many factors are associated with seniors’ willingness for institutional care. As mentioned above, marital status is one of the important predictors. It’s also found that age, chronic diseases, depression, perceived family harmony, knowledge about elder care institutions, and economic status were associated with seniors’ willingness for institutional care [6, 13]. Many studies indicated that seniors with older age were more willing for institutional care [14, 15]. Those who had a positive attitude towards institutional care were more willing to enter a nursing home [16]. Low income level was negatively associated with a willingness to stay in an elder home [2]. An analysis of longitudinal data on Finnish older adults also showed that the probability of admission to long-term institutional care was inversely associated with household income [17].

Gender is a significant factor determining long-term institutional care [14]. Some studies find that older women were more likely to enter institutional care than older men [11, 18]. Gender difference towards institutional care reflect the difference not only in demographic characteristics but also in socioeconomic circumstances, living arrangements and health conditions. Compared with men, women have worse health-related quality of life (HRQL) among elderly. It’s found that gender is not only a determinant for poorer physical health-related quality of life but also for poorer mental health-related quality of life among those seniors aged 65 years or older [19, 20]. However, no studies have examined gender disparity towards institutional care willingness among single seniors in rural China. To fill this gap in literature, the present study aims to examine the gender difference in the utilization willingness of institutional care among the single seniors in rural China. To do so, we have following specific objectives. First, we will estimate the utilization willingness of institutional care among the single seniors in rural China. Second, we will examine the gender difference in use willingness of institutional care. Third, we will identify the factors associated with utilization willingness of institutional care in male and female single seniors respectively.

## Methods

### Settings and participants

This study was conducted in Shandong Province. There were about 97 million people in Shandong province in 2012, among which the seniors accounted for over 15% [21]. In this study, a 3-stage cluster sampling was used to select participants (See the Additional file 1 the flow chart of the sampling procedure). Firstly, all districts and counties in Shandong Province were stratified into three groups on the ground of GDP per capita (2011) separately. Secondly, we chose one district and one county from each group. Thus, three urban districts (Huaiyin, Dongchangfu and Zhangdian) and three rural counties (Qufu, Chiping and Leling) were chosen as the study sites. Similarly, we then chose three sub-districts and three townships in each sampling district or county on the basis of GDP per capita. Lastly, three communities and three villages were selected from each chosen sub-district and township. Therefore, we selected 27 urban communities and 27 rural villages in total. The study was initially designed to enroll 470 single seniors, a sample size that was considered sufficient with an assumption of 5.0% of rural single seniors were willing for institutional care, and an estimated odds ratio (male versus female) of 3, with an alpha of 0.05 and a power of 0.90. Finally, a total of 505 single older people are included in the analysis.

### Data collection

Data were collected from November 2011 to January 2012 by using a house-to-house interview. Face-to-face interviews were conducted among the elderly using a structured questionnaire by trained master students from Shandong University School of Public Health. To ensure quality, completed questionnaires were carefully checked by quality supervisors at the end of each day. The questionnaire included demographic characteristics, living arrangement of the households, relationship with children, marital status, economic status, psychological well-being and willingness for institutional care. The total Cronbach’s α coefficient of the entire questionnaire is 0.841, indicating a high reliability.

### Variables and Measures

#### Independent variable

The independent variable was seniors’ willingness for institutional care, which was evaluated on the ground of interviewees’ answers to ‘which way of elder care are you willing for?’ If the response was ‘institutional care’, the willingness for institutional care could be coded as ‘yes’. On the contrary, if the answer was ‘home-based care’ and ‘others’, willingness for institutional care would be categorized as ‘no’.

#### Dependent variables

Sociodemographic characteristics, including age (60-, 70-, and 80+ years), gender (male vs. female), education (illiteracy, primary school, and junior school or above), past occupation (farmer vs. others), number of children (≤3 vs. >3), relationship with children (good vs. normal or bad), residence (urban vs. rural), living arrangements (living alone, living with children or others), and household income (Q1, Q2, Q3 and Q4). Quartile 1 (Q1) is the poorest and Quartile 4 (Q4) is the richest. Family size is a continuous variable and it represents the number of family members.

Physical health status, including self-rated health status (good vs. normal or bad), non-communicable chronic diseases (NCDs) in the past six months (yes vs. no), and activity of daily living (ADL). ADL is assessed by ADL instrument which is consisted of Physical Self-maintenance Scale and Instrumental Activities of Daily Living Scale designed by Lawton and Brody [22]. ADL Scale is used to evaluate people’s simple and basic ability to practice one’s normal life independently. The reliability and validity of ADL instrument in Chinese-language version was verified to be good [23]. Scores of ADL can be divided into 3 levels, the higher level represents more severe dysfunction. Level 1, 2, and 3 means mild dysfunction, moderate dysfunction, and severe dysfunction respectively.

Psychological well-being includes social support, and psychological stress. Social support was assessed on the basis of Social Support Revalued Scale (SSRS) designed by Xiao and its reliability and validity has been demonstrated to be good [24]. Higher scores means more social support. Psychological stress was evaluated on the ground of 10-item Kessler Scale (K10). K10 is an effective tool to assess people’s psychological status designed by Kessler, Mroczek and so on [25]. The Chinese-language version of K10 has been verified to be of good reliability and validity [26].

### Statistical Analysis

The data was double entered and checked using EpiData 6.04. Statistical analyses were performed using SPSS 21.0. For continuous variables, p value was calculated using Student *t* test; for categorical variables, *p* value was calculated using chi-square test. Binary logistic regression with an enter method was employed to assess the association of willingness for institutional care with gender. All reported CIs were calculated at the 95% level. Statistical significance was set at the 5% level.

## Results

### Basic information of the participants

Table 1 shows basic information about the 505 seniors. About 5.7% rural single seniors had willingness for institutional care, and 3.5% females and 9.3% males had institutionalization willingness separately. Generally speaking, the majority of the elderly were female (38.2%), at the ages of 60 to 69 (45.1%), illiterate or semiliterate (70.1%), farmers (91.9%), having 3 or less children (60.2%), having good relationship with children (87.9%), having normal or bad self-reported health status (52.5%), having mild dysfunction (50.7%), having NCDs (71.3%) and the poorest (45.0%). The single elderly’s K10 score was 17.5 ± 7.3 and their per-capita living space was 40.9 ± 38.2. Age (*P* = 0.026), education (*P* = 0.000), past occupation (*P* = 0.001), living arrangements (*P* = 0.000), number of children (*P* = 0.000), and ADL (*P* = 0.001) were significantly different between rural single males and females.

**Table 1:** Socio-demographic characteristics of rural single seniors in Shandong, China, 2012

Characteristics	Total^a^ n (%)	Rural single male n (%)	Rural single female n (%)	*χ* ^*2*^/t	*p*
Observations	505(100)	193(38.2)	312(61.8)		
Age				7.307	**0.026**
60-	228(45.1)	98(50.8)	130(41.7)		
70-	200(39.6)	62(32.1)	138(44.2)		
80-	77(15.2)	33(17.1)	44(14.1)		
Education				82.226	**0.000**
Illiteracy or semiliterate	354(70.1)	90(46.6)	264(84.6)		
Primary school	110(21.8)	74(38.3)	36(11.5)		
Junior school or above	41(8.1)	29(15.0)	12(3.8)		
Past occupation				11.998	**0.001**
Farmer	464(91.9)	167(86.5)	297(95.2)		
Others	41(8.1)	26(13.5)	15(4.8)		
Living arrangements				33.787	**0.000**
Alone	156(30.9)	70(36.3)	86(27.6)		
With children or others	349(69.1)	123(63.7)	226(72.4)		
Number of children				13.747	**0.000**
0-3	304(60.2)	136(70.5)	168(53.8)		
> 3	201(39.8)	57(29.5)	144(46.2)		
Relationship with children				0.225	0.635
Good	444(87.9)	168(87.0)	276(88.5)		
Normal or bad	61(12.1)	25(13.0)	36(11.5)		
Social support	29.5 ± 6.3	28.5 ± 7.0	30.2 ± 5.8	32.880	0.328
Self-reported health status				3.179	0.075
Good	240(47.5)	82(42.5)	158(50.6)		
Normal or bad	265(52.5)	111(57.5)	154(49.4)		
Psychological stress	17.5 ± 7.3	17.1 ± 7.3	17.7 ± 7.2	24.587	0.699
ADL^b^				13.963	**0.001**
I	256(50.7)	117(60.6)	139(44.6)		
II	134(26.5)	36(18.7)	98(31.4)		
III	115(22.8)	40(20.7)	75(24.0)		
NCD^c^				0.274	0.601
Yes	360(71.3)	135(69.9)	225(72.1)		
No	145(28.7)	58(30.1)	87(27.9)		
Income^d^				4.200	0.241
Q1	227(45.0)	83(43.0)	144(46.2)		
Q2	129(25.5)	58(30.1)	71(22.8)		
Q3	105(20.8)	39(20.2)	66(21.2)		
Q4	44(8.7)	13(6.7)	31(9.9)		
Per-capita living space	40.9 ± 38.2	45.4 ± 49.6	38.1 ± 28.8	86.844	0.713

^a^Rural singles include those who are unmarried(10.5%), divorced(1.2%), widowed(86.5%), separated(1.8%)

^b^ADL means activity of daily living

^c^NCD means non-communicable chronic diseases

^d^Quartile 1 (Q1) is the poorest and Quartile 4 (Q4) is the richest

### Association of gender and willingness for institutionalization among single seniors

We presented our results in two models so that we could better understand the association between gender and institutionalization willingness in rural single seniors (Table 2). Model 1 showed that institutionalization willingness was statistically lower in single females than in single males (OR = 0.36; 95CI 0.16-0.77). When other variables were controlled, as shown in Model 2, willingness for institutionalization was still statistically lower in single females than in single males (OR = 0.19; 95 CI 0.06-0.57).

**Table 2:** Association of gender and willingness for institutionalization in single seniors in rural Shandong, China,2012

Characteristics	Model 1 (No covariates)		Model 2 (Covariates)	
	OR (95% CI)	*P*	OR (95% CI)	*P*
Gender				
Male	1.0		1.0	
Female	0.36(0.16-0.77)	**0.009**	0.19(0.06-0.57)	**0.003**
Age				
60-			1.0	
70-			0.53(0.19-1.43)	0.208
80-			0.000(0.000)	0.996
Education				
Illiteracy or semiliterate			1.0	
Primary school			0.77(0.27-2.23)	0.628
Junior school or above			0.08(0.01-1.07)	0.056
Past occupation				
Farmer			1.0	
Others			0.42(0.07-2.65)	0.354
Living arrangements				
Along			1.0	
With children or others			0.13(0.03-0.52)	**0.004**
Number of children				
≤ 3			1.0	
> 3			0.67(0.21-2.12)	0.495
Relationship with children				
Good			1.0	
Normal or bad			1.96(0.68-5.67)	0.214
Social support			1.02(0.94-1.10)	0.643
Self-reported health status				
Good			1.0	
Normal or bad			1.26(0.41-3.86)	0.693
Psychological stress			1.10(1.03-1.18)	**0.005**
ADL^a^				
I			1.0	
II			2.27(0.80-6.41)	0.123
III			0.59(0.15-2.36)	0.457
NCD^b^				
Yes			1.0	
NO			1.22(0.40-3.75)	0.724
Income^c^				
Q1^a^			1.0	
Q2			0.79(0.24-2.57)	0.692
Q3			0.27(0.03-2.51)	0.248
Q4			1.77(0.16-19.24)	0.641
Per-capita living space			1.00(0.98-1.02)	0.983

Model 1, Model 2: Association of gender differences and willingness for institutionalization in single seniors in rural Shandong, China (*n* = 505)

^a^ADL means activity of daily living

^b^NCD means non-communicable chronic diseases

^c^Quartile 1 (Q1) is the poorest and Quartile 4 (Q4) is the richest

### Factors associated with institutionalization willingness among single male seniors

Table 3 showed factors associated with willingness for institutional care among single older males in rural areas. Univariate analysis indicated that those single older males who lived with children or others had less willingness for institutionalization than those who lived alone (*P* = 0.002). It also showed that those single males who had higher social support (*P* = 0.008) were less willing for institutional care. Those who had higher psychological stress (*P* = 0.014) were more likely to prefer institutional care. Multi-logistic analysis identified that those who lived with children or others (*P* = 0.032) were less willing for institutional care than those who lived alone. Those who had higher psychological stress (*P* = 0.045) were more willing for institutional care.

**Table 3:** Factors associated with institutionalization willingness among single male seniors in rural Shandong, China,2012 (*n* = 193)

Characteristics	Willingness for institutionalization		OR_c_ (95% CI)	*P*	OR_a_ (95% CI)	*p*
	Yes (%)	No (%)				
*n* = 193	18(9.3)	175(90.7)				
Age					NA	
60-	13(13.3)	85(86.7)	1.0			
70-	5(8.1)	57(91.9)	0.57(0.19-1.70)	0.315		
80-	0(0.0)	33(100.0)	0.000(0.000)	0.998		
Education					NA	
Illiteracy or semiliterate	11(12.2)	79(87.8)	1.0			
Primary school	6(8.1)	68(91.9)	0.63(0.223-1.80)	0.393		
Junior school or above	1(3.4)	28(96.6)	0.26(0.03-2.08)	0.202		
Past occupation					NA	
Farmer	16(9.6)	151(90.4)	1.0			
Others	2(7.7)	24(92.3)	0.79(0.17-3.64)	0.759		
Living arrangements						
Alone	13(18.6)	57(81.4)	1.0		1.0	
With children or others	5(4.1)	118(95.9)	0.19(0.06-0.55)	**0.002**	0.26(0.08-0.89)	**0.032**
Number of children					NA	
≤ 3	16(11.8)	120(88.2)	1.0			
> 3	2(3.5)	55(96.5)	0.27(0.06-1.23)	0.090		
Relationship with children					NA	
Good	15(8.9)	153(91.1)	1.0			
Normal or bad	3(12.0)	22(88.0)	1.39(0.37-5.20)	0.624		
Social support	18(9.3)	175(90.7)	0.91(0.85-0.98)	**0.008**	0.96(0.89-1.04)	0.318
Self-reported health status					NA	
Good	5(6.1)	77(93.9)	1.0			
Normal or bad	13(11.7)	98(88.3)	2.04(0.70-5.98)	0.192		
Psychological stress	18(9.3)	175(90.7)	1.08(1.02-1.14)	**0.014**	1.06(1.00-1.13)	**0.045**
ADL^a^					NA	
I	10(8.5)	107(91.5)	1.0			
II	5(13.9)	31(86.1)	1.73(0.55-5.43)	0.350		
III	3(7.5)	37(92.5)	0.87(0.23-3.32)	0.836		
NCD^b^					NA	
Yes	15(11.1)	120(88.9)	1.0			
NO	3(5.2)	55(94.8)	0.44(0.12-1.570)	0.204		
Income^c^					NA	
Q1	12(14.5)	71(85.5)	1.0			
Q2	4(6.9)	54(93.1)	0.44(0.13-1.43)	0.173		
Q3	1(2.6)	38(97.4)	0.16(0.020-1.24)	0.079		
Q4	1(7.7)	12(92.3)	0.49(0.06-4.15)	0.515		
Per-capita living space	18(9.3)	175(90.7)	1.00(0.10-1.01)	0.448	NA	

OR_c_: crude odds ratio

OR_a_ : adjusted odds ratio

^a^ADL means activity of daily living

^b^NCD means non-communicable chronic diseases

^c^Quartile 1 (Q1) is the poorest and Quartile 4 (Q4) is the richest

### Factors associated with institutionalization willingness among single female seniors 

As shown in Table 4, univariate analysis showed that those single female seniors in rural areas who lived with children or others (*P* = 0.001) were less willing for institutional care than those who lived alone. Those rural single female seniors who had normal or bad relationship with children (*P* = 0.000), who had higher psychological stress (*P* = 0.001), who had moderate dysfunction (*P* = 0.041), who had more per-capita living space (*P* = 0.020) had more willingness for institutionalization than those who had good relationship with children, who were less psychologically stressful, who had mild dysfunction or who had less per-capita living space. Similar with the result of single male seniors, multi-logistic regression indicated that those single female seniors who lived with children or others (*P* = 0.002) were less willing for institutional care. Those with higher psychological stress (*P* = 0.013) were more likely to prefer institutional care.

**Table 4:** Factors associated with institutionalization willingness among single female seniors in rural Shandong, China, 2012 (*n* = 312)

Characteristics	Willingness for institutionalization		OR_c_ (95% CI)	p	OR_a_ (95%CI)	p
	Yes (%)	No (%)				
*n* = 312	11(3.5)	301(96.5)				
Age					NA	
60-	5(3.8)	125(96.2)	1.0			
70-	6(4.3)	132(95.7)	1.14(0.34-3.82)	0.836		
80-	0(0.0)	44(100.0)	0.000(0.000)	0.998		
Education					NA	
Illiteracy or semiliterate	9(3.4)	255(96.6)	1.0			
Primary school	2(5.6)	34(94.4)	1.67(0.35-8.04)	0.525		
Junior school or above	0(0.0)	12(100.0)	0.000(0.000)	0.999		
Past occupation					NA	
Farmer	11(3.7)	286(96.3)	1.0			
Others	0(0.0)	15(100.0)	0.000(0.000)	0.999		
Living arrangements						
Alone	10(11.6)	76(88.4)	1.0		1.0	
With children or others	1(0.4)	225(99.6)	0.03(0.00-0.27)	**0.001**		**0.002**
Number of children					NA	
≤ 3	7(4.2)	161(95.8)	1.0			
> 3	4(2.8)	140(97.2)	0.66(0.19-2.30)	0.510		
Relationship with children						
Good	5(1.8)	271(98.2)	1.0		1.0	
Normal or bad	6(16.7)	30(83.3)	10.84(3.12-37.66)	**0.000**	4.49(0.92-20.35)	0.051
Social support	11(3.5)	301(96.5)	0.91(0.83-1.01)	0.082	NA	
Self-reported health status					NA	
Good	3(1.9)	155(98.1)	1.0			
Normal or bad	8(5.2)	146(94.8)	2.83(0.74-10.88)	0.130		
Psychological stress	11(3.5)	301(96.5)	1.15(1.06-1.24)	**0.001**	1.14(1.03-1.27)	**0.013**
ADL^a^						
I	2(1.4)	137(98.6)	1.0		1.0	
II	7(7.1)	91(92.9)	5.27(1.07-25.93)	**0.041**	4.08(0.63-26.19)	0.139
III	2(2.7)	73(97.3)	1.88(0.260-13.60)	0.533	0.57(0.05-5.90)	0.635
NCD^b^					NA	
Yes	7(3.1)	218(96.9)	1.0			
NO	4(4.6)	83(95.4)	1.50(0.43-5.26)	0.526		
Income^c^					NA	
Q1	9(6.3)	135(93.8)	1.0			
Q2	2(2.8)	69(97.2)	0.44(0.09-2.07)	0.295		
Q3	0(0.0)	66(100.0)	0.000(0.000)	0.997		
Q4	0(0.0)	31(100.0)	0.000(0.000)	0.998		
Per-capita living space	11(3.5)	301(96.5)	1.02(1.00-1.03)	**0.020**	1.00(0.98-1.03)	0.723

OR_c_: crude odds ratio

OR_a_ : adjusted odds ratio

^a^ADL means activity of daily living

^b^NCD means non-communicable chronic diseases

^c^Quartile 1 (Q1) is the poorest and Quartile 4 (Q4) is the richest

## Discussion

Our study found that about 5.7% rural single seniors were willing for institutional care and this was lower than the 45% found in a study of the elderly of a similar age in Korean American elders [27]. It was lower than 16.7% found in a study of the population aged 65 or over in Taiwan, China [16]. It was also lower than the 9.69% found in older population in Zhejiang, China [28] and 44.8% found in a study of the elderly of a similar age in Chengdu, China [29]. Compared with above mentioned studies, our study focuses on rural single seniors’ willingness for institutional care. Such population has its own specific characteristics. Rural single seniors mostly have lower income and rely more on their children. Consideration of entering institutional care might make them feel abandoned by their own children [13]. Shandong, compared with above mentioned places, is rather a conservative province which is deeply affected by Confucianism^1^. Confucianism emphasize that children should undertake the responsibility of taking care of older parents [30]. Thus, many adult children would choose to take care of senior adults at home rather than sending them to nursing homes to avoid moral condemnation. Older people nowadays are affected by Confucianism which makes them feel better to be taken care of at home rather than in nursing homes [31]. If they were sent to nursing homes, they might feel being abandoned and considered their children disobedient. The culture of filial piety is profoundly rooted in Shandong people’s mind. All these differences might explain the variation in our study and the ones quoted above.

We found that there was a significant gender difference towards institutionalization willingness among rural single seniors in China. This was consistent with another study which indicated that rural women were less willing for institutional care [6]. One study found that husbands’ probability of nursing home admission doubled following spousal loss while this probability of nursing home entry among women was unchanged after spousal death [32]. A study by Luppa, M. et al also found an increased probability of NHP (nursing home placement) if the person was unmarried (including single, widowed or divorced) and/or living alone, but with a higher effect for males than for females [33]. Wives often provide more informal care to their spouse than husbands do. If an old man was unmarried, they might seek help through other ways such as institutional care. For women, they get less informal care from their husbands, if they were widowed or divorced, they might be less willing for institutional care than men. Informal care, particularly by the family, is the most important source of care for most elderly people. Traditionally, women were the primary caregivers for husbands and children, especially in the context of Confucian thought. Women undertook more responsibility of taking care of the family while men were responsible for feeding their family. Women’s care for their husbands was actually an informal care which was a substitute for formal care in an institution. Thus, those male single seniors would tend to be more willing for institutional care if they were widowed, divorced or unmarried.

In this study, rural seniors who lived with children or others were found to be less willing for institutional care both for single males and females, which was consistent with previous studies [28, 34]. Single seniors who lived alone would easily feel lonely and might be more eager for communication. This might be why those single seniors, both for men and women, would prefer institutional care. Some studies have found that a great number of family members, particularly adult children, usually means more financial and physical assistance for parents [35]. Due to one-child policy announced in 1970s, however, family size has shrunk which made traditional familial care more unavailable. With the increasing amount of young people moving to cities and leaving their older parents, the family support system for seniors, especially for those single seniors, is becoming a challenging issue, which deserves addressing.

Similarly, psychological stress was found to be associated with utilization willingness of institutional care for both single males and females, which was in agreement with previous studies [34]. To avoid excessive reliance on family members which may result in tensions in family, when seniors had psychological stress, they would rather choose institutional care [36]. In addition, institutional care organizations can provide health care which might benefit the seniors physically and emotionally [37]. Those single seniors with higher psychological stress could get better health services, especially mental health services, in old-age care institutions. Better health services would increase the probability of their willingness for institutional care.

This study has some limitations. Firstly, our study has a cross-sectional design and it could only interpret the association of institutionalization willingness and gender difference instead of casual relationships. Secondly, all data were based on self-reported measures which could lead to several forms of bias.

## Conclusion

In summary, our study found that single males were more willing for institutional care than single females in rural China, which might be due to the responsibility that the women undertake at home. It was also found that psychological stress was a major determinant for institutionalization willingness in both single males and single females in rural areas. The seniors who lived alone were more willing to institutional care for both rural single males and females compared with those lived with children or others. Targeted policies should be made for different subgroups of rural single seniors, thus, appropriate institutional care can be offered.

## Additional file

Additional file 1: Sampling procedure. (DOCX 21 kb)

## Abbreviations

ADL: Activity of daily living
GDP: Gross domestic products
K10: Kessler 10
NCDs: Non-communicable chronic diseases

## Footnotes

1 Confucianism, also known as Ruism, is described as tradition, a philosophy, a humanistic religion, with particular emphasis on the importance of the family and social harmony, rather than on an otherworldly source of spiritual values. The core of Confucianism is humanistic.
