# Supplementary material for: Gender difference in utilization willingness of institutional care among the single seniors: evidence from rural Shandong, China
Source: Int J Equity Health. 2017 May 12;16:77. doi: 10.1186/s12939-017-0577-z (PMC5427541; doi:10.1186/s12939-017-0577-z)
Supplement: Additional file 1: — Sampling procedure. (DOCX 21 kb) [file 12939_2017_577_MOESM1_ESM.docx]

***Supplementary File 1:* Sampling procedure**

All districts and counties in Shandong province were stratified into three groups on the ground of GDP per capita.

3 urban districts and three rural counties were chosen as the study sites. We then chose 3 sub-districts and 3 townships in each sampling district or county on the basis of GDP per capita.

3 communities and 3 villages were selected from each chosen sub-district and township. In total, 27 urban communities and 27 rural villages were selected.

In the analysis segment, we only include those seniors who are single in rural areas. Thus, 505 single seniors are analyzed in our article.
